# Epstein–Barr virus tegument protein BGLF2 in exosomes released from virus-producing cells facilitates de novo infection

**DOI:** 10.1186/s12964-022-00902-7

**Published:** 2022-06-21

**Authors:** Yoshitaka Sato, Masahiro Yaguchi, Yusuke Okuno, Hanako Ishimaru, Ken Sagou, Somi Ozaki, Takeshi Suzuki, Tomoki Inagaki, Miki Umeda, Takahiro Watanabe, Masahiro Fujimuro, Takayuki Murata, Hiroshi Kimura

**Affiliations:** 1 Department of Virology, Nagoya University Graduate School of Medicine, Nagoya, Japan; 2 PRESTO, Japan Science and Technology Agency (JST), Kawaguchi, Japan; 3 Department of Virology, Graduate School of Medicine, Nagoya City University, Nagoya, Japan; 4 Department of Cell Biology, Kyoto Pharmaceutical University, Kyoto, Japan; 5 Department of Hematology and Oncology, Nagoya University Graduate School of Medicine, Nagoya, Japan; 6 Department of Dermatology, School of Medicine, University of California Davis (UC Davis), Sacramento, CA USA; 7 Department of Virology and Parasitology, Fujita Health University School of Medicine, Toyoake, Japan; 8 Present Address: Division of Clinical Virology, Center for Infectious Diseases, Kobe University Graduate School of Medicine, Kobe, Japan

**Keywords:** Epstein–Barr virus, Exosome, BGLF2, De novo infection, Extraviral particle

## Abstract

**Background:**

Viruses must adapt to the environment of their host cells to establish infection and persist. Diverse mammalian cells, including virus-infected cells, release extracellular vesicles such as exosomes containing proteins and miRNAs, and use these vesicles to mediate intercellular communication. However, the roles of exosomes in viral infection remain unclear.

**Results:**

We screened viral proteins to identify those responsible for the exosome-mediated enhancement of Epstein–Barr virus (EBV) infection. We identified BGLF2 protein encapsulated in exosomes, which were released by EBV-infected cells. BGLF2 protein is a tegument protein that exists in the space between the envelope and nucleocapsid, and it is released into the cytoplasm shortly after infection. BGLF2 protein-containing exosomes enhanced viral gene expression and repressed innate immunity, thereby supporting the EBV infection.

**Conclusions:**

The EBV tegument protein BGLF2 is encapsulated in exosomes and released by infected cells to facilitate the establishment of EBV infection. These findings suggest that tegument proteins support viral infection not only between the envelope and nucleocapsid, as well as in extraviral particles such as exosomes.

**Graphical abstract:**

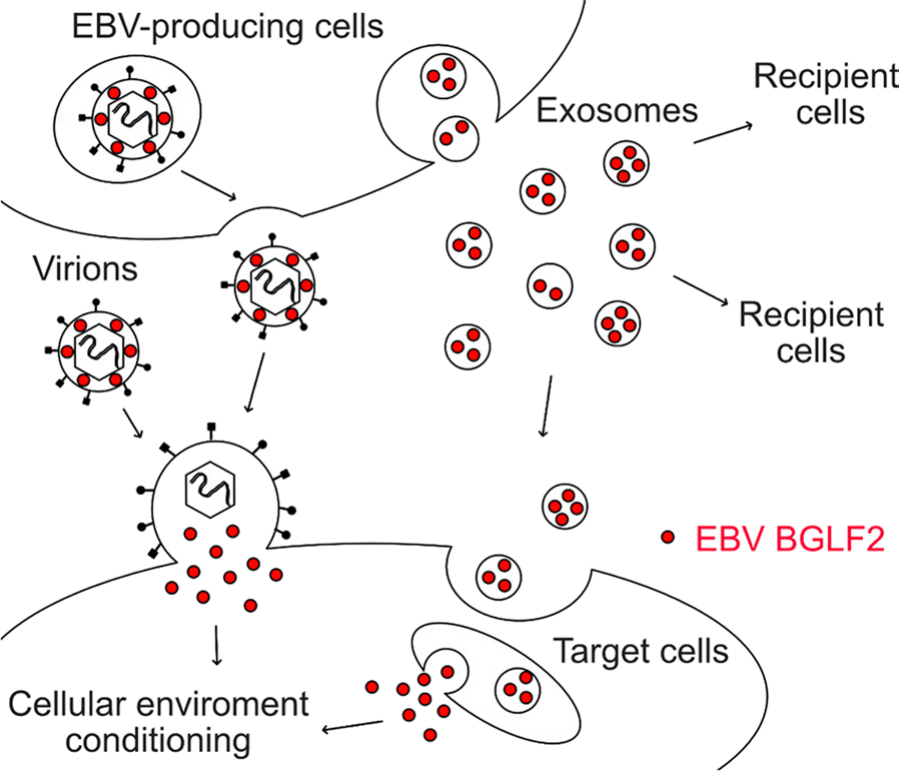

**Video abstract**

**Supplementary Information:**

The online version contains supplementary material available at 10.1186/s12964-022-00902-7.

## Background

Epstein–Barr virus (EBV), a member of the herpesvirus family, mainly infects B cells. Approximately 90% of the world’s population is asymptomatically infected by EBV, but the virus can cause infectious mononucleosis and malignancies such as Burkitt lymphoma, T/NK cell lymphoma, nasopharyngeal carcinoma, and gastric carcinoma [1, 2]. EBV displays a biphasic life cycle including a latent phase and a lytic (productive replication) phase [3]. Although EBV preferentially remains latent in host cells without viral production after primary infection, a limited number of viral genes are expressed [4]. Under certain circumstances, latency is disrupted, and the virus shifts to the lytic phase. During this productive replication phase, all (> 80) viral genes are expressed, the viral genome undergoes replication, and progeny virions are produced [5, 6].

Similar to that of other herpesviruses, the EBV virion is composed of a viral genomic DNA-containing nucleocapsid surrounded by a viral envelope with glycoprotein spikes on its surface [7, 8]. Located between the nucleocapsid and outer viral envelope is the viral tegument, which includes > 10 viral proteins called tegument proteins [9] that are involved in the morphogenesis, envelopment, and egress of progeny virions and primary de novo infection [10]. Because they are released shortly after infection into the cytoplasm, tegument proteins play major roles in infectivity by modulating the intracellular environment. Indeed, we recently revealed that the EBV tegument protein BGLF2 increases viral infectivity [11].

Extracellular vesicles such as exosomes are released by eukaryotic cells. These vesicles can contain proteins, RNA, miRNA, or DNA, and they serve as crucial mediators of intercellular communication involved in organismal homeostasis and disease states [12–14]. The roles of extracellular vesicles have received considerable attention in recent years. Exosomes secreted by virus-infected cells contain viral materials [15]. Interestingly, EBV-infected cell lines secrete a higher number of extracellular vesicles than uninfected cells [16], leading us to hypothesize that EBV utilizes exosomes to influence infection and pathogenesis by facilitating viral replication or activating immune evasion strategies. For instance, the viral oncoprotein latent membrane protein 1 (LMP1) has been detected in exosomes [17–19]. LMP1-positive exosomes activate the extracellular signal-regulated kinase and phosphoinositide 3-kinase (PI3K)/Akt signaling pathways in the recipient cells [20]. Although EBV carries more than 80 genes, many of which are lytic proteins, the majority of viral proteins detected in exosomes are latent proteins [21], as these latent proteins have been identified according to the phenotypes of EBV-associated tumors. Therefore, it remains unclear which EBV lytic proteins are encapsulated in exosomes and function during infection.

In this study, we investigated the role of EBV protein-containing exosomes during infection. We performed a series of screenings and identified the tegument protein BGLF2, which enhanced de novo infection via exosomes. Our results suggest that this tegument protein functions via extracellular vesicle transfer similarly as virus particles, ensuring efficient and robust modulation of the microenvironment for the infection.

## Results

### *Screening for EBV proteins that modulate infection *via* exosomal transfer*

**Fig. 1:**
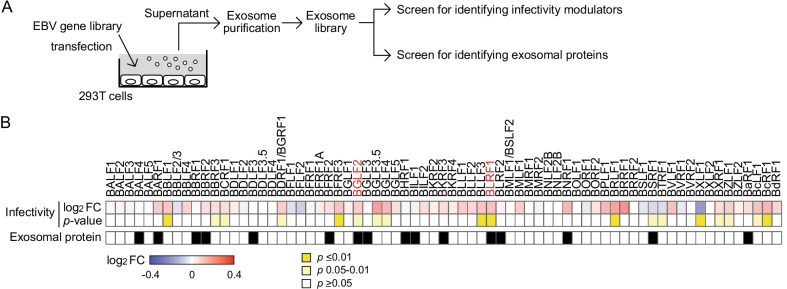
Combined screenings for the viral proteins that regulate the infectivity via exosomal transfer. **A** The workflow of the screenings. Exosomes were purified from the conditioned media of HEK293T cells that were transfected with each plasmid expressing an Epstein–Barr virus (EBV) gene. To analyze modulators of infectivity, EBV-negative Akata(-) cells were pretreated with purified exosomes and then infected with EGFP-EBV. The infectivity was determined at 48 h post-infection (hpi) by FACS analysis. To identify exosomal proteins, purified exosomes were analyzed by immunoblotting with an anti-HA antibody. **B** Heatmap presenting the fold change (FC) of infectivity of EBV with Akata(-) cells in the presence of each exosome at 48 hpi. Infectivity changes (top panel) reflect the log_2_ FC relative to the control exosome purified from empty vector-transfected cells. FCs greater than 1 are displayed in red; and thoses smaller than 1 are displayed in blue. *p* values are presented in the middle panel. The viral protein in purified exosomes is indicated in black in the bottom panel

To explore the roles of exosomes containing EBV proteins during infection, we generated an exosome library from 293 T cells expressing 71 different epitope-tagged EBV lytic proteins [11], and screened for EBV genes that modulated infection and that were detected in exosomes (Fig. 1A). EBV-negative Akata(-) B-cells treated with each exosome from the library were experimentally infected with EBV, and the infectivity at 48 h post-infection (hpi) was compared (Fig. 1B, upper panel). Additionally, the exosome library was subjected to immunoblotting using an anti-hemagglutinin (HA) antibody to detect EBV proteins in the exosomes (Fig. 1B, lower panel). By integrating multiple screening strategies, we found BGLF2 and BLRF1 within exosomes, observing that they enhanced EBV infection. Because BLRF1 encodes envelope glycoprotein N, which localized to the membrane, we focused on the tegument protein BGLF2.

### Exosomal BGLF2 exerts its function in recipient cells

**Fig. 2:**
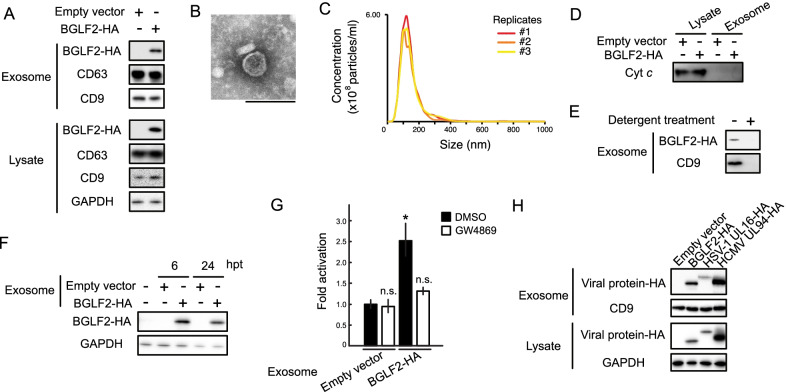
Functional BGLF2 proteins are transferred to the recipient cells via exosomes. **A** BGLF2 protein-containing exosomes were released by BGLF2-expressing cells. HEK293T cells were transfected with HA-tagged BGLF2-expression or empty plasmid. Exosomes were isolated from the cell supernatant. CD63 and CD9 served as exosome markers. **B** TEM image of the exosomes purified from BGLF2-expressing HEK293T cells. Scale bar represents 100 nm. **C** NanoSight profiles displaying size distribution for exosomes purified from BGLF2-expressing HEK293T cells. **D** Western blotting results showing the absence or underrepresentation of the negative exosome marker cytochrome-C (Cyt *c*) in isolated exosomes. **E** BGLF2 protein was incorporated into exosomes. Exosomes were purified from supernatant treated with or without 0.25% Triton-X 100 for 5 min at room temperature. **F** BGLF2 was delivered to the recipient cells via exosomes. HEK293T cells were treated with BGLF2-containing exosomes for the indicated number of hours, and then washed extensively, and harvested. Cell lysates were analyzed by immunoblotting. **G** BGLF2 transferred via exosomes activated the AP-1 promoter. Exosomes were purified from HEK293T cells transfected with BGLF2-expression plasmid or empty plasmid in the presence or absence of GW4869. HEK293T cells transfected with the pAP-1-Luc and promoterless-Rluc plasmids, and then treated with BGLF2-containing exosomes. Cell lysates were obtained at 24 h after treatment and analyzed using luciferase reporter assays. Values (mean ± SEs) were calculated from three independent experiments. Asterisks, *p* < 0.05; n.s., not significant. **H** The orthologs of BGLF2 are incorporated into exosomes. HEK293T cells were transfected with expression plasmids containing HA-tagged BGLF2 or its HSV-1 or HCMV orthologs. Exosomes were isolated from the cell supernatant

Because exosomes were purified by a precipitation-based method in our screening for easy handling, we could not dismiss the possibility of non-exosome contamination [22]. To examine the incorporation of BGLF2 into the exosomes, we isolated exosomes from the culture supernatant of 293 T cells expressing BGLF2-HA using an affinity-based method. BGLF2 was also detected in exosomes purified using both exosome-affinity resin (Fig. 2A) and anti-CD9 antibody (data not shown). The size range and morphology of exosomes were characterized via transmission electron microscopy (TEM) and nanoparticle tracking analysis (NTA), respectively (Fig. 2B and C). We confirmed that cellular cytochrome-C [23] was not detected in the purified exosomes, suggesting no contamination by other types of extracellular vesicles (Fig. 2D). Moreover, we demonstrated that detergent treatment of the culture supernatant impeded the detection of BGLF2 in the exosomes, suggesting that BGLF2 was encapsulated within the exosomes (Fig. 2E).

BGLF2 stimulates the activator protein 1 (AP-1) signaling pathway [11, 24]. To assess the function of BGLF2-containing exosomes, we added them to cells transfected with AP-1 reporter (pAP-1-Luc) and control plasmids. As shown in Fig. 2F, exosomal BGLF2 was taken up by the recipient cells, and treatment with BGLF2-containing exosomes enhanced AP-1 reporter activity (Fig. 2G). GW4869, which blocks exosome release by inhibiting neutral sphingomyelinase [25], prevented AP-1 activation (Fig. 2G). These findings indicate that functional BGLF2 is transferred via exosomes to recipient cells. BGLF2 exhibited a pan-cellular localization pattern without a restricted colocalization with CD63, a marker for multivesicular bodies (Additional File 1: Figure S1), consistent with previous reports [26, 27],

All herpesviruses possess a BGLF2 homolog [24]. Therefore, we tested whether the orthologs of EBV BGLF2 such as herpes simplex virus type 1 (HSV-1) UL16 and human cytomegalovirus (HCMV) UL94 proteins were incorporated into exosomes. HSV-1 UL16 and HCMV UL94 were detected in exosomes purified from cells expressing HSV-1 UL16 and HCMV UL94 (Fig. 2H). These findings indicate a conserved role of exosomes containing BGLF2 or its herpesvirus ortholog proteins.

### Extravirion particle BGLF2 enhances the infectivity of BGLF2-knockout (BGLF2-KO) EBV

**Fig. 3:**
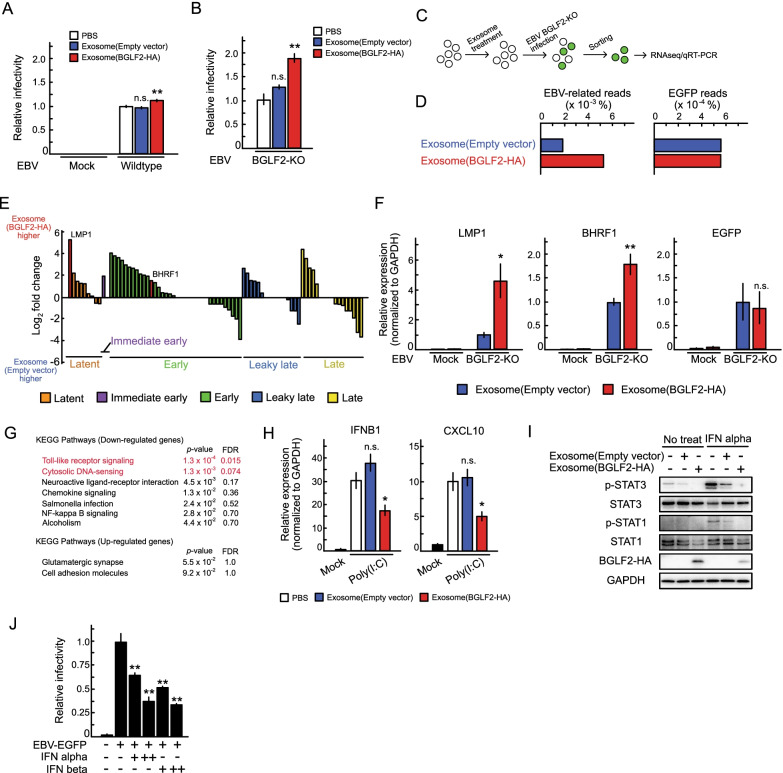
Exosomal BGLF2 enhances the infectivity of EBV. **A** and **B** Akata(-) cells were infected with wildtype (**A**) and BGLF2-KO EBV (**B**) in the presence of BGLF2-containing exosomes. After 2 days, GFP positivity was determined by FACS. Results are presented as the mean ± SE of at least three independent experiments and as the relative infectivity to control treatment with PBS (infectivity value of 1). Double asterisks, *p* < 0.01; n.s., not significant. **C** The workflow of sample collection for RNA-seq/qRT-PCR analyses. Akata(-) cells were pretreated with exosomes for 16 h, and then infected with BGLF2-KO EBV. The infected cells were collected via FACS at 24 hpi. Total RNA extracted from the sorted cells was subjected to RNA-seq and qRT-PCR. **D** Read counts of EBV genes in BGLF2-KO EBV-infected cells that were treated with BGLF2-containing or control exosomes by RNA-seq. **E** Gene expression levels were normalized as counts per million followed by log_2_-transformation with a pseudo-count of 1. Each bar indicates the log_2_ fold change of a viral gene expression between treatment with exosomes with or without BGLF2. Viral gene expression kinetics are categorized into five groups: latent, immediate early, early, leaky late, and late [53]. **F** Validation of enhanced EBV gene expression by qPCR. Data are presented as the mean ± SE. Samples were tested in duplicate. Asterisk, *p* < 0.05; double asterisks, *p* < 0.01; n.s., not significant. **G** DAVID analysis of RNA-seq data from BGLF2-KO EBV-positive cells treated with BGLF2-containing or control exosomes. FDR, false discovery rate. **H** HEK293 cells were treated with BGLF2-containing exosomes for 16 h. Cells were challenged with transfection of poly(I:C) (2 μg/ml) for 2.5 h. RT-qPCR was conducted. Data are presented as the mean ± SE of three independent experiments. Asterisk, *p* < 0.05; n.s., not significant. **H** Exosomal BGLF2 inhibited type I IFN signaling. AGS/EBV-EGFP cells were pretreated with exosomes for 16 h, and then infected with or without IFN alpha (1000 Unit/mL) for 1 h. Lysates were analyzed by immunoblotting using the indicated antibodies. BGLF2-HA was detected using the anti-HA antibody. **J** Type I IFN inhibited EBV infection. Akata(-) cells were infected with EBV-EGFP in the presence of IFN alpha and beta (100 or 500 Unit/mL). After 2 days, GFP positivity was determined by FACS. Results are presented as the mean ± SE of three independent experiments. Double asterisks, *p* < 0.01; n.s., not significant

Next, we examined whether exosomal BGLF2 supported EBV infection. Treatment with exosomes containing BGLF2 slightly, but statistic-significantly, enhanced the infectivity of wild-type EBV compared with the effect of a control treatment (Fig. 3A). Because the virus solution used in this study was contaminated by exosomes released from virus-producing cells, we used BGLF2-KO EBV to observed clear effects of BGLF2-containing exosomes. BGLF2-KO reduced EBV infectivity upon the de novo infection of B-cells (Additional File 2: Figure S2), consistent with a previous report [11]. Exosomal BGLF2 significantly enhanced the infectivity of BGLF2-KO EBV (Fig. 3B). To evaluate the effect of BGLF2-containing exosomes on EBV infection, we performed RNA-seq analysis (Fig. 3C). Akata(-) cells were pre-treated with BGLF2-containing exosomes or control exosomes and then infected with BGLF2-KO EBV. We collected infected cells by fluorescence-activated cell sorting (FACS) at 24 hpi according to EGFP-positivity, and performed RNA-seq to obtain transcriptome information. As shown in Fig. 3D and E, treatment with BGLF2-containing exosomes induced the expression of EBV genes, although the expression level of EGFP, which was driven from a eukaryotic promoter [28], was unchanged. This enhanced EBV mRNA expression was validated using quantitative polymerase chain reaction (qPCR), whereas no viral gene expression was detected in the absence of EBV infection (Fig. 3F). LMP1 was one of the most significantly expressed viral genes between the groups treated with exosomes with or without BGLF2 (Fig. 3E).

Among genes downregulated following treatment with BGLF2-containing exosomes, the terms “Toll-like receptor signaling” and “cytosolic DNA-sensing” were enriched in DAVID analysis [29] of RNA-seq data (Fig. 3G). This finding is consistent with a recent report that ORF33, the ortholog of BGLF2 in Kaposi’s sarcoma-associated herpesvirus (KSHV), suppresses the interferon (IFN) beta production pathway for immune evasion [30]. Consequently, we investigated whether BGLF2-containing exosomes modulated the innate immune response. Polyinosinic-polycytidylic acid [poly(I:C)] was used to stimulate the TLR3-mediated IFN beta production pathway [31]. As shown in Fig. 3H, BGLF2-containing exosomes inhibited IFN beta 1 (IFNB1) and its downstream effector, C-X-C motif chemokine ligand (CXCL) 10 mRNA expression under poly(I:C) stimulation. We further evaluated the effects of exosomal BGLF2 on type I IFN signaling. Treatment of BGLF2-containing exosomes reduced the levels of phosphorylated signal transducer and activator of transcription (STAT) 1 (p-STAT1), and p-STAT3 in IFN alpha-treated AGS/EBV-EGFP cells (Fig. 3I), consistent with a previous observation in the cells that overexpressing BGLF2 [32]. We only observed a minor reduction of STAT1 and STAT3 phosphorylation in the cells treated with control exosomes compared to the findings in PBS-treated cells, implying the ability of exosomes to antagonize cytokines [33, 34]. We also confirmed that type I IFNs inhibited EBV infection in a dose-dependent manner (Fig. 3J).

### EBV-infected cells release BGLF2-containing exosomes during the productive replication

**Fig. 4:**
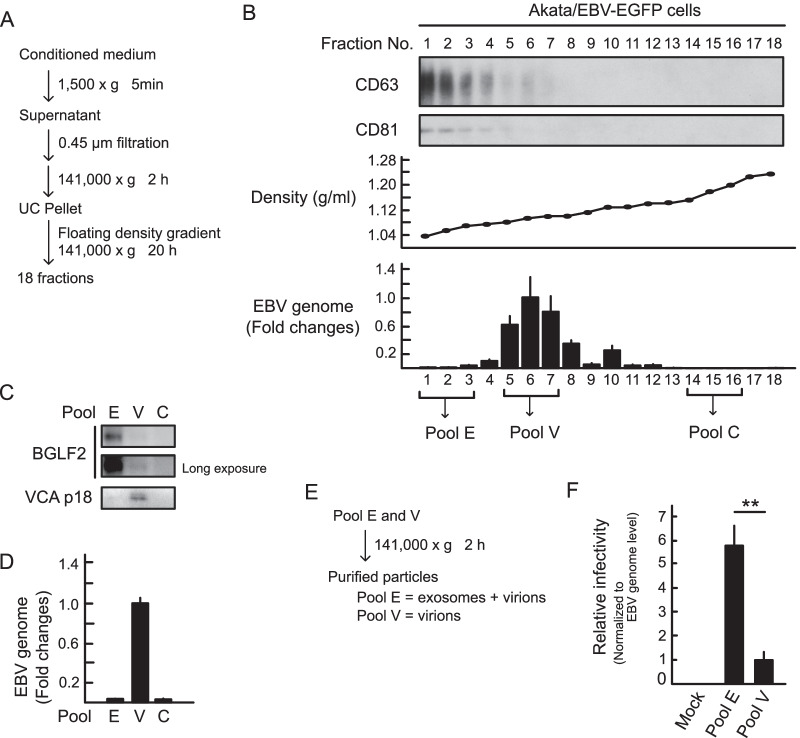
BGLF2-containing exosomes are released by infected cells. **A** Centrifugation protocol and workflow for separation and enrichment of exosomes and virions. **B** Exosome marker proteins were enriched in fractions #1-3 after iodixanol floating density gradient centrifugation. For immunoblotting, proteins in each fraction were concentrated by acetone precipitation. CD63 and CD81 served as exosome markers. The EBV genome in each fraction was quantified by qPCR. **C** and **D** Pool E (fractions #1-3) containing the BGLF2 protein. Each pool (E, fractions #1-3; V, fractions #5-6; and C, fractions #14-16) was analyzed by immunoblotting (**C**) and qPCR (**D**). Data are presented as the mean ± SE. Viral capsid antigen (VCA) p18 served as a virion marker. **E** Workflow for particle purification. **F** Akata(-) cells were incubated with purified particles for 24 h. GFP positivity was determined by FACS. The EBV genome of each pool was quantified by qPCR. The results are presented as the mean ± SE of four independent experiments after normalization to the EBV genome levels and as relative infectivity to pool V (infectivity value of 1). Double asterisks, *p* < 0.01

We investigated whether BGLF2-containing exosomes could be released from infected cells. Conditioned medium from lytic-induced Akata/EBV-EGFP cells by anti-human IgG was subjected to floating density gradient ultracentrifugation and separated into 18 fractions (Fig. 4A). Each fraction was characterized by immunoblotting with exosome marker antibodies and qPCR analysis for EBV genome detection. The peak of the exosome markers probably represented exosomes and separated from a peak of the viral genome that represented virion particles (Fig. 4B). Because the antibody against BGLF2 did not exhibit high sensitivity, three fractions mainly containing exosomes (pool E), virions (pool V), and control (pool C) were pooled, and further evaluated. As shown in Figs. 4C, a large portion of BGLF2 protein was detected in pool E, which showing that viral capsid antigen (VCA) was below the limit of detection, suggesting that infected cells released BGLF2-containing exosomes during lytic replication. However, we could not completely separate exosomes from virions (Fig. 4D). To assess the impact of exosomes released from the lytic-induced cells on EBV infection, we purified the particles from pools E and V (Fig. 4E). Akata(-) cells were incubated with these particles and GFP positivity was measured by FACS. Of note, the particles purified from pool E contained lytic-associated exosomes and a small number of virions. As shown in Fig. 4F, the infectivity per viral genome of pool E was higher than that of pool V, suggesting that lytic-associated exosomes facilitated the infectivity of EBV.

**Fig. 5:**
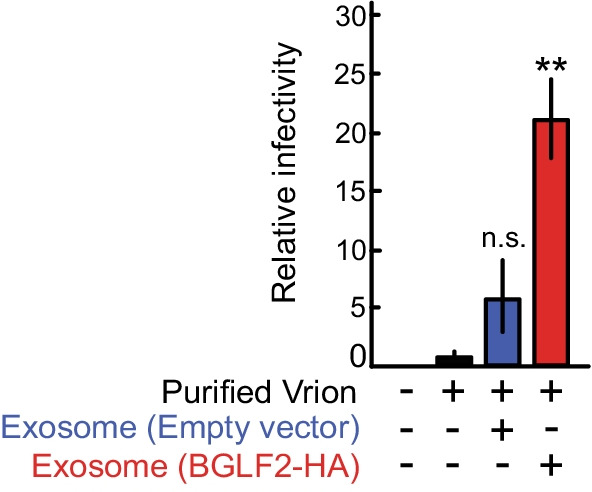
BGLF2-containing exosomes enhance the infectivity of EBV. Akata(-) cells were infected with purified virions from pool V in the presence of BGLF2-containing exosomes. GFP positivity was determined by FACS at 24 h post-infection. Results are presented as the mean ± SE of four independent experiments and as relative infectivity to control treatment with PBS (infectivity value of 1). Double asterisks, *p* < 0.01; n.s., not significant

To further determine whether BGLF2-containing exosomes could enhance EBV infection, we used the particles purified from pool V as pure virions, because pool V contained a large amount of the EBV genome (Fig. 4D). Akata(-) cells were pretreated with BGLF2-containing exosomes or control exosomes, which were prepared from 293 T cells, and then incubated with purified virions from pool V. As shown in Fig. 5, exosomal BGLF2 significantly enhanced the infectivity of EBV.

Together, our data indicate that BGLF2 is encapsulated in viral particles and exosomes released by infected cells. BGLF2 delivered via exosomes promotes optimal EBV infection by enhancing viral gene expression and repressing innate immunity.

## Discussion

Virally infected cells release virions as infectious particles and non-infectious particles. All herpesvirus-infected cells are known to release L-particles in addition to virions [35–37]. L-particles are non-infectious because they consist of the virus envelope and tegument proteins while lacking a viral genome and nucleocapsid. This conserved release of L-particles may shed light on an essential role of non-infectious particles in creating a microenvironment that facilitates their replication, spread, and persistence in the host cells. It was recently demonstrated that HSV-1 L-particles mediate immune evasion by downregulating CD83 on mature dendritic cells [38].

BGLF2 protein impairs type I IFN signaling through Tyk2 [32] and dampens NF-κB activity [39] in a cell-dependent manner during lytic infection. Expression of KSHV ORF33 (the ortholog of BGLF2) suppresses the IFN beta production pathway [30]. In this study, we demonstrated that the exosomal tegument protein BGLF2 was transferred to the target cells, in which it in turn enhanced EBV infectivity by manipulating the cellular environment. BGLF2 delivered via exosomes increased the expression of EBV genes (Figs. 3D–F). LMP1 is upregulated through the binding of AP-1 with CCAAT enhancer binding proteins on its promoter [40]. The EBV gene BHRF1 is highly expressed during the first 2 days after infection [41, 42]. These findings imply a role for BGLF2 in viral gene expression in infected cells, consistent with previous reports that BGLF2 activates p38, JNK, and AP-1 to induce viral gene expression [11, 24].

Exosomal BGLF2 failed to completely restore the reduced titer of BGLF2-KO EBV to the WT level (Fig. 3 and Additional File 2: Figure S2). However, we could not dismiss the possibility that the WT virus solution contained BGLF2-carrying and other exosomes carrying EBV proteins and miRNA released from virus-producing cells. BGLF2-containing exosomes clearly potentiated the infectivity of purified virions albeit under the optimized condition (Fig. 5). These findings highlight the role of exosomal BGLF2 protein in EBV infection.

Furthermore, exosomes prepared from BGLF2-expressing 293 T cells inhibited IFN beta production stimulation (Fig. 3H), protecting infected cells from a type I IFN-mediated response during de novo infection. Exosomal BGLF2 protein was functional in recipient cells (Figs. 2G and 3) suggesting that exosomes serve as a carrier of herpesvirus tegument proteins to target cells. Our findings using exosomes prepared from BGLF2-expressing 293 T cells indicate that BGLF2 protein is transferred via exosomes into recipient cells, in which they subsequently adjust the cellular condition at multiple levels. However, we cannot completely dismiss the possibility that a factor other than BGLF2 itself is responsible for this modulation in the cells, because of technical limitations. Further study with technical improvements such as a single-molecule tracking or a whole-content analysis of exosomes is required to investigate this possibility.

BGLF2-containing exosomes were released from EBV-infected cells that lytically produced infectious virions (Fig. 4). EBV can hijack and use extracellular vesicles such as exosomes. It is known that EBV increases the extracellular vesicle secretion [16], and EBV miRNAs delivered via exosomes increase the severity of EBV-mediated lymphoproliferative disease [43]. Although exosomes are released in exocytic bursts upon fusion of the multivesicular bodies to the cell membrane [44], the final envelopment site for EBV is the Golgi apparatus [45], indicating that these non-infectious particles (L-particles and exosomes) are generated in non-Golgi compartments. Furthermore, BGLF2 and its orthologs in HSV-1 and HCMV were incorporated into exosomes (Fig. 2H), indicating that manipulation of the cellular environment by exosomes is a conserved mechanism among herpesvirus. The results of this study also suggest a previously unforeseen essential role for non-infectious particles in viral protection.

## Conclusion

Both virions and exosomes containing BGLF2 tegument protein were released by EBV-infected cells. Exosomal BGLF2 enhanced the EBV infection by upregulating EBV gene expression and suppressing type I IFN production. These findings suggest that exosomes containing the viral protein cooperate with virions to establish EBV infection.

## Methods

### Cells, plasmids, and reagents

HEK293T (ATCC CRL-3216) and HEK293/EBV(dBGLF2) cells [11] were maintained in DMEM supplemented with 10% fetal bovine serum (FBS). Akata(-) [46], and Akata/EBV-EGFP [47] cells were cultured in RPMI1640 medium containing 10% FBS. AGS/EBV-EGFP cells (a kind gift from Hironori Yoshiyama) [48] were grown in F-12 HAM’s medium supplemented with 10% FBS and 400 μg/mL G418.

The BZLF1-expression plasmid and HA-tagged EBV lytic protein-expression library were described previously [11, 49]. To express C-terminal HA-tagged HSV-1 UL16 and HCMV UL94, constructs expressing UL16 and UL94 in pcDNA3 (Thermo Fisher Scientific, Waltham, MA, USA) were prepared by PCR and the In-Fusion cloning system (Takara Bio, Kusatsu, Shiga, Japan). The inserted DNA sequence of each vector was confirmed by direct DNA sequencing. The reporter plasmid pAP1-Luc and promoterless-RLuc (pGL4.70) were purchased from Promega (Madison, WI, USA).

Bortezomib (Cat# A10160; AdooQ BioScience, Irvine, CA, USA) was used at a concentration of 100 nM. Recombinant human type I IFN alpha (Cat# 11200-1) and IFN beta (Cat# 11415-1) were purchased from PBL Assay Science (Piscataway, NJ, USA). For inhibition of exosome secretion, cells were treated with 10 μM GW4869 (Cat# 13127; Cayman Chemical, Ann Arbor, MI, USA). Transient transfection of poly(I:C) (Cat# 4287/10; R&D Systems, Minneapolis, MN, USA) was performed using Lipofectamine 2000 (Thermo Fisher Scientific) according to the manufacturer’s protocol.

### Antibodies and immunoblotting

Anti-HA (3F10) rat antibody was purchased from Sigma-Aldrich (St. Louis, MO, USA). Anti-CD63 (Ts63) mouse and anti-VCA (PA1-73003) goat antibodies were obtained from Thermo Fisher Scientific. Rabbit anti-GAPDH (14C10), rabbit anti-CD9 (D8O1A), rabbit anti-STAT3 (79D7), rabbit anti-phospho-STAT3 (D3A7), rabbit STAT1 (#9172), rabbit anti-phospho-STAT1 (Tyr701) and horseradish peroxidase-conjugated secondary antibodies were purchased from Cell Signaling Technology (Danvers, MA, USA). Anti-CD81 (5A6) mouse antibody was obtained from Santa Cruz Biotechnology (Dallas, TX, USA). Antiserum against EBV BGLF2 was prepared by immunizing a rabbit with the synthetic peptide NH2-CAHVNILRGWTEDDSPGTS-COOH, and the serum was purified by affinity purification using the same peptide (Cosmo Bio, Tokyo, Japan). Immunoblot and signal detection were performed as described previously [49].

### Virus preparation

EGFP-EBV was obtained from the 8-day-old cell-free supernatant of AGS/EGFP-EBV cells. The cell-free supernatant was filtered through 0.45 μm filters (Merck, Darmstadt, Germany) and then used as a virus stock. To prepare BGLF2-KO virus, HEK293/EBV(dBGLF2) cells [11] were transfected with BZLF1 expression plasmid using Lipofectamine 2000 (Thermo Fisher Scientific) according to the manufacturer’s instructions. Cells and their media were harvested and freeze-thawed, and cell debris was removed. The supernatant after centrifugation was filtered through 0.45 μm filters and then used as a virus stock. EBV-negative Akata(-) cells were infected with the virus, and EGFP-positive cells were counted by FACS to measure the viral titer.

### Exosome purification and treatment

Exosome-containing conditioned medium was centrifuged at 2000 × g to remove cells. Exosomes were purified with a Total Exosome Isolation Kit (Thermo Fisher Scientific) for screening or an exoEasy Maxi Kit (Qiagen, Hilden, Germany) according to the manufacturer’s protocols.

Size and particle number were analyzed using the LM10 or NS300 nanoparticle characterization system (NanoSight; Malvern Panalytical, Worcestershire, United Kingdom). Cells were treated with exosomes at 6 × 10^3^ particles/cell.

### TEM

For negative staining prior to TEM analysis, the samples were absorbed on formvar film coated copper grids, and stained with 2% phosphotungstic acid solution (pH 7.0) for 1 min. The grids were observed using a transmission electron microscope (JEM-1400Plus; JEOL Ltd., Tokyo, Japan) at an acceleration voltage of 100 kV. Digital images were captured using a CCD camera (EM-14830RUBY2; JEOL Ltd.).

### Floating density gradient ultracentrifugation

Semi-confluent Akata/EBV-eGFP cells were washed with PBS twice and suspended in medium containing EV-free FBS and anti-human IgG to induce lytic replication. After 72 h, 100 ml of the conditioned medium were centrifuged at 1500 × g and 4 °C for 5 min to remove cells and debris. The supernatant was filtered through 0.45-μm filters and extracellular vesicles including exosomes and virions were pelleted by ultracentrifugation at 141,000 × g at 4 °C for 2 h in a swinging-bucket rotor (SW28; Beckman Coulter, Brea, CA, USA). The pellet was resuspended in filtered PBS and centrifuged at 18,300 × g and 4 °C for 10 min to remove insoluble aggregates. The supernatant was mixed with iodixanol (Optiprep; Cat#1114542, Cosmo Bio) (40% (w/v) final concentration of iodixanol). A discontinuous iodixanol gradient was prepared as follows: sample/iodixanol was applied to the bottom of the tube, followed by 35, 30, 25, and 20% (w/v) iodixanol/PBS, and covered by 15% (w/v) iodixanol/PBS. The density gradient centrifugation was performed at 141,000 × g and 4 °C for 20 h in a SW28 swing rotor (Beckman Coulter). In total, 18 fractions were collected starting from the top.

To purify virion particles from pooled fraction, pool V was diluted in PBS and ultracentrifuged at 141,000 × g and 4 °C for 2 h in a SW32Ti swing rotor (Beckman Coulter). The pellet was resuspended in filtered PBS.

### Screening

HEK293T cells were transfected with each plasmid expressing an EBV gene using Lipofectamine 2000 (Thermo Fisher Scientific). On the following day, the medium was changed to DMEM supplemented with 5% exosome-depleted FBS (System Biosciences, Palo Alto, CA, USA). After 48 h, exosomes were purified from the conditioned medium. To identify exosomal proteins, we analyzed purified exosomes by immunoblotting. Immunoblotting and signal detection were performed as described previously [50]. Exosomal proteins were determined by comparing the expression of positive (LMP1-contining exosomes) and negative (exosomes isolated from the cells transfected with empty vector) controls. To analyze modulators of infectivity, EBV-negative Akata(-) cells were treated with purified exosomes in RPMI1640 supplemented with 5% exosome-depleted FBS (System Biosciences, Palo Alto, CA, USA). After 6 h, cells were infected with EGFP-EBV. At 48 h after infection, cells were fixed with 4% paraformaldehyde. The infectivity was determined using a flow cytometer (FACS Canto, BD Biosciences, San Jose, CA, USA).

### Luciferase reporter assay

HEK293T cells were transfected with AP-1 reporter plasmid (pAP1-Luc) and promoterless-RLuc as an internal control using Lipofectamine 2000 (Thermo Fisher Scientific) and incubated in DMEM supplemented with 5% exosome-depleted FBS. After 48 h, cells were treated with purified exosomes. Cells were lysed after 24 h and subjected to luciferase assays using the Dual-Luciferase Reporter Assay System (Promega).

### RNA-seq

Akata(-) cells were pretreated with BGLF2-containing exosomes or control exosomes for 16 h, and then infected with BGLF2-KO EBV at room temperature for 2 h with agitation. After infection, cells were resuspended in medium containing exosomes. The top 30,000 EBV-infected cells expressing EGFP were sorted by a FACS Aria II Cell Sorter (BD Biosciences) at 24 hpi.

Total RNA was extracted using an RNeasy Mini kit (Qiagen). Evaluation of RNA, RNA-seq library preparation, Illumina sequencing, and data preprocessing were performed as described previously [51].

### qPCR

Total RNA was reverse-transcribed to cDNA using a PrimeScript II Reverse transcriptase kit (Takara Bio) or the *SuperPrep* II Cell Lysis & RT kit for qPCR (TOYOBO, Osaka, Japan). To detect the viral genome in virions, samples were treated with DNase I (NEB) to remove non-capsidated viral genomes. Viral DNA was purified using a QIAamp DNA blood mini kit (QIAGEN). Viral DNA and mRNA levels were analyzed by qPCR using the 7500 Fast DX Real-Time PCR system (Applied Biosystems, Foster City, CA, USA) as described previously [19]. The primer sequences used in this study are listed in Table 1. Other primer information was described previously [52].

**Table 1:** Oligonucleotide primers used for qPCR

Primer name	Sequence (5′ to 3′)
BALF2 forward	GCCCGTCCGGTTGTCA
BALF2 reverse	GGCCACGCTGATAAAGTTGTC
BHRF1 forward	AAATGGTACCCTGCATCCTG
BHRF1 reverse	CCACATGTTCGGTGTGTGTT
LMP1 forward	CTGATGATCACCCTCCTGCT
LMP1 reverse	CTAAGACAAGTAAGCACCCGAAG
EGFP forward	ACGTAAACGGCCACAAGTTC
EGFP reverse	AAGTCGTGCTGCTTCATGTG
GAPDH forward	CCTCCAAGGAGTAAGACCCC
GAPDH reverse	TGTGAGGAGGGGAGATTCAG

### Statistical analysis

Results are presented as the means ± standard error (SE) of at least three independent experiments. Statistical analyses were performed using Microsoft Excel. Welch’s *t*-test was used to determine significance, and *p* < 0.05 indicated statistical significance.

### Data availability

All RNA-seq datasets have been deposited to the DNA Data Bank of Japan (DDBJ; https://www.ddbj.nig.ac.jp/index-e.html) under the accession number: DRA010388.

## Supplementary Information

**Additional file 1. Fig S1.** Cellular localization of BGLF2 and CD63 in HEK293 cells. HEK293 cells were transfected with HA-tagged BGLF2-expression plasmid. Cells were fixed at 2 days post-transfection and then stained with anti-HA and anti-CD63 antibodies.

**Additional file 2. Fig. S2**. Comparison of the infectivity between wildtype and BGLF2-KO EBV. Akata(-) cells were infected with wildtype and BGLF2-KO EBV. After 2 days, GFP positivity was determined by FACS. Results are presented as the mean ± SE of three independent experiments and as the relative infectivity to BGLF2-KO EBV (infectivity value of 1).

## Data Availability

All data generated or analysed during this study are included in this published article.

## Abbreviations

EBV: Epstein–Barr virus
AP-1: Activator protein-1
Cyt c: Cytochrome-C
FACS: Fluorescence-activated cell sorting
HA: Hemagglutinin
HCMV: Human cytomegalovirus
HSV-1: Herpes simplex virus type 1
IFN: Interferon
KO: Knockout
KSHV: Kaposi’s sarcoma-associated herpesvirus
LMP1: Latent membrane protein 1
NTA: Nanoparticle tracking analysis
PI3K: Phosphoinositide 3-kinase
poly(I:C): Polyinosinic-polycytidylic acid
qPCR: Quantitative polymerase chain reaction
TEM: Transmission electron microscopy
