# Supplementary figures and images for: Epstein–Barr virus tegument protein BGLF2 in exosomes released from virus-producing cells facilitates de novo infection
Source: Cell Commun Signal. 2022 Jun 21;20:95. doi: 10.1186/s12964-022-00902-7 (PMC9210680; doi:10.1186/s12964-022-00902-7)

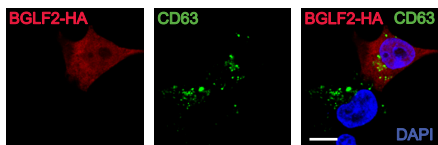

Supplement: Supplementary file 2 — Additional file 1. Fig S1. Cellular localization of BGLF2 and CD63 in HEK293 cells. HEK293 cells were transfected with HA-tagged BGLF2-expression plasmid. Cells were fixed at 2 days post-transfection and then stained with anti-HA and anti-CD63 antibodies. [file 12964_2022_902_MOESM2_ESM.pdf]

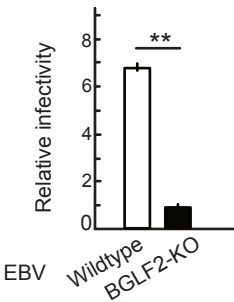

Supplement: Supplementary file 3 — Additional file 2. Fig. S2. Comparison of the infectivity between wildtype and BGLF2-KO EBV. Akata(-) cells were infected with wildtype and BGLF2-KO EBV. After 2 days, GFP positivity was determined by FACS. Results are presented as the mean ± SE of three independent experiments and as the relative infectivity to BGLF2-KO EBV (infectivity value of 1). [file 12964_2022_902_MOESM3_ESM.pdf]
